# Bronchoscopic Local Nd:YAG Laser Hyperthermia in the Treatment of Lung Cancer

**DOI:** 10.1155/DTE.1.159

**Published:** 1995

**Authors:** Ryosuke Ono

**Affiliations:** Department of Endoscopy National Cancer Center Hospital, 1-1 Tsukiji 5-Chome Chuo-Ku Tokyo 104 Japan

## Abstract

Between March 1989 and July 1990, 13 patients with tracheobronchial cancer were treated by laser hyperthermia at the National Cancer Center Hospital. A complete response was achieved in 9 patients (with 9 carcinoma lesions), with no evidence of local recurrence on follow-up ranging from 4 to 29 months. Four patients had a partial response, requiring alternative therapy.

The surface area of the lesions showing complete response was less than 3 cm^2^. These lesions had a superficial appearance by bronchoscopic observation. Our experience suggests that laser hyperthermia may be a useful alternative to surgical resection in patients with small localized tumors confined within the tracheobronchial wall.


*Diagnostic and Therapeutic Endoscopy*, 1995, Vol. 1, pp. 159–164
Reprints available directly from the publisher
Photocopying permitted by license only

© 1995 Harwood Academic Publishers GmbH
Printed in Malaysia

### Bronchoscopic Local Nd:YAG Laser Hyperthermia in the Treatment of Lung Cancer

RYOSUKE ONO

*Department of Endoscopy, National Cancer Center Hospital, 1-1, Tsukiji 5-Chome, Chuo-Ku, Tokyo, 104 JAPAN*

*(Received September 27, 1993; in final form, August 15, 1994)*

Between March 1989 and July 1990, 13 patients with tracheobronchial cancer were treated by laser hyperthermia at the National Cancer Center Hospital. A complete response was achieved in 9 patients (with 9 carcinoma lesions), with no evidence of local recurrence on follow-up ranging from 4 to 29 months. Four patients had a partial response, requiring alternative therapy.

The surface area of the lesions showing complete response was less than 3 $cm^2$. These lesions had a superficial appearance by bronchoscopic observation. Our experience suggests that laser hyperthermia may be a useful alternative to surgical resection in patients with small localized tumors confined within the tracheobronchial wall.

**KEY WORDS:** ND:YAG Laser, Flexible Bronchoscope with 2 Channels, Malignant Tumor of Tracheobronchial Tree, Thermister

### INTRODUCTION

Hyperthermia therapy has been performed clinically in combination with radiotherapy for cancer. Interstitial hyperthermia in combination with interstitial radiotherapy is gaining acceptance rapidly as an effective treatment for recurrent and/or persistent malignant tumors. Local tumor control of more than 50% in previously treated patients and a low incidence of treatment complications (1–3) has established the clinical usefulness of the technique.

In recent years, contact irradiation by Nd:YAG laser using a ceramic tip has been performed more frequently than noncontact irradiation for treating cancer in the gastrointestinal tract. The tip is inserted into a cancerous lesion and the temperature increased to 42° to 45° at a relatively low laser power level (3–5W) for 15 to 30 minutes, causing tumor destruction.

The temperature range is controlled by a computer system. This method of laser hyperthermia can be clinically applied more safely than ordinary vaporizing laser irradiation not only for radical treatment of early cancer, but also for recanalization of obstructions caused by advanced cancer in the gastrointestinal tract. The effect of laser hyperthermia is increased when applied in combination with radiotherapy.

Therapeutic options for treatment of early-stage lung cancer of the hilar type traditionally have been limited to surgical resection. The introduction of laser hyperthermia has provided a new therapeutic alternative to surgery. This form of therapy may be particularly helpful, because patients with bronchogenic carcinoma are at risk for the development of a subsequent primary lung cancer. Therapy that potentially preserves lung parenchyma would be of benefit in the long-term management of these patients. The following is a brief report on the role of laser hyperthermia in the treatment of roentgenographically occult carcinoma of the tracheobronchial tree.

### MATERIALS AND METHODS

### Selection of Patients

All patients included in our study had biopsy-proved malignant tumors involving the tracheobronchial tree. The condition of each patient was evaluated by a bronchoscopist, a thoracic surgeon, and an oncologist. Severe reduction in pulmonary and/or cardiac function precluded surgical resection in each patient. Two operable patients refused surgery, and they elected to receive laser hyperthermia. Informed

Address for Correspondence: Ryosuke Ono, M.D., Department of Endoscopy, National Cancer Center Hospital, 1-1, Tsukiji 5-Chome, Chuo-Ku, Tokyo, 104 Japan.

consent was obtained from each patient. The individual reasons for patient selection are shown in Table 1.

### Source of Light for Irradiation

A medical-grade quartz optical fiber, 10 m long with a core diameter of 400 μm was used to transmit the laser beam through the bronchoscope to the site of endobronchial cancer. The output beam diverged approximately 100° to 120°. The fiber was passed through the working channel of the flexible fiberoptic bronchoscope (Olympus BF2TR) so that the distal end protruded from the tip of the bronchoscope.

Laser light was transmitted to the tumor in all cases by means of intralesional implantation of the fiber probe. The distal tip of the optical fiber was attached to a 3-mm cone-shaped ceramic probe. To keep a distance between the cone rod probe and thermister, they were inserted through separate channels of a double channel bronchoscope, as shown in Figure 1.

The thermister was placed 5 mm from the treatment site to keep the temperature of the lesion at 42°C ± 1.0°C, and a period of 20 to 30 minutes was set as one course of treatment. The laser beam initially was in continuous wave mode until the temperature reached 42°C, at which point the mode automatically changed to pulse wave mode.

In this way, an intense spot with a diameter of 10 mm could be obtained. The light dose rate was estimated in terms of power density during interstitial(i) treatment (in W/cm). The delivered dose was the total dose delivered interstitially (i) (in Joules/cm). This value was estimated to be the total amount of light delivered to the whole area.

The proximal end of the optical fiber was attached to a positioning device that was coupled to a continuous wave or pulse wave Nd:YAG laser (Pentax Model SLY-2). The wave length produced with the Nd:YAG laser was 1015 nm in all patients. Laser pulses were delivered for 0.1 to 9.9 seconds at 2 to 100 W/pulse.

The thermister containing a thermometer was 2.2 mm in diameter and was situated in the apex of the apparatus. The thermister thermometer signal was transmitted to the thermocontroller to allow function with the shutters on/off, on the basis of thermosettlement. This would allow beam regulation for the inner part of the tumor. The thermal recorder recorded temperature as a graph. The thermister thermometer was passed through the open channel of the flexible fiberoptic bronchoscope so that the distal end protruded from the tip of the bronchoscope.

**Table 1** Individual reasons for patients selection

| *Reason* | *No. of Patients* |
|---|---|
| Poor respiratory function | 4 |
| Cardiac disease | 4 |
| Multiple primary malignant lesions | 3 |
| Surgical operation refused and they selected laser hyperthermia | 2 |
| Total: | 13 |

### Treatment

All patients were orally intubated over a flexible fiberoptic bronchoscope, and 2% lidocaine anesthesia was administered to the lower airways as needed. Photographs were taken before treatment and at the time of each subsequent bronchoscopic procedure. Either biopsy or brushing was performed several days before the treatment.

For the tumors treated with intralesional insertion of the fiber probe, the corresponding ranges were 2 to 3 W and 1003 to 3832 J/cm.

The selection of power and energy density was initially based on early clinical data from intralesional laser treatment of metastatic skin lesions. The data reported by Tajiri et al and Suzuki et al from in vivo and in vitro animal experiments influenced us to reduce power densities to decrease the possibility of hyperthermic effects (5, 6). Tumors with substantial bulk that protruded into the lumen were treated with intralesional insertion of the laser fiber. Since this study most patients had lesions with protrusion into the lumen, therefore, only intralesional insertion of the laser fiber was used.

### Follow-Up and Interpretation of Results

All the patients understood the need for close follow-up, including multiple bronchoscopic procedures, to maintain the therapeutic response. We routinely did a clean-up bronchoscopic procedure 1 or 2 days after each therapeutic session. The patients also underwent bronchoscopy at 2 to 3 month intervals for at least 3 years after demonstration of an initial complete response.

The patients were classified as having a complete response if on follow-up evaluation for 4 weeks or longer, no tumor was found on chest roentgenogram and bronchoscopy with negative biopsy and brushings. A partial response was defined as a persistent tumor on follow-up chest roentgenography or bronchoscopic examination.

### RESULTS

During this study, 13 patients received laser hyperthermia at National Cancer Center Hospital. All except one had squamous cell carcinoma. One patient was a woman, and the other 12 patients were men (age range, 36–79 years; mean, 64.7 years).

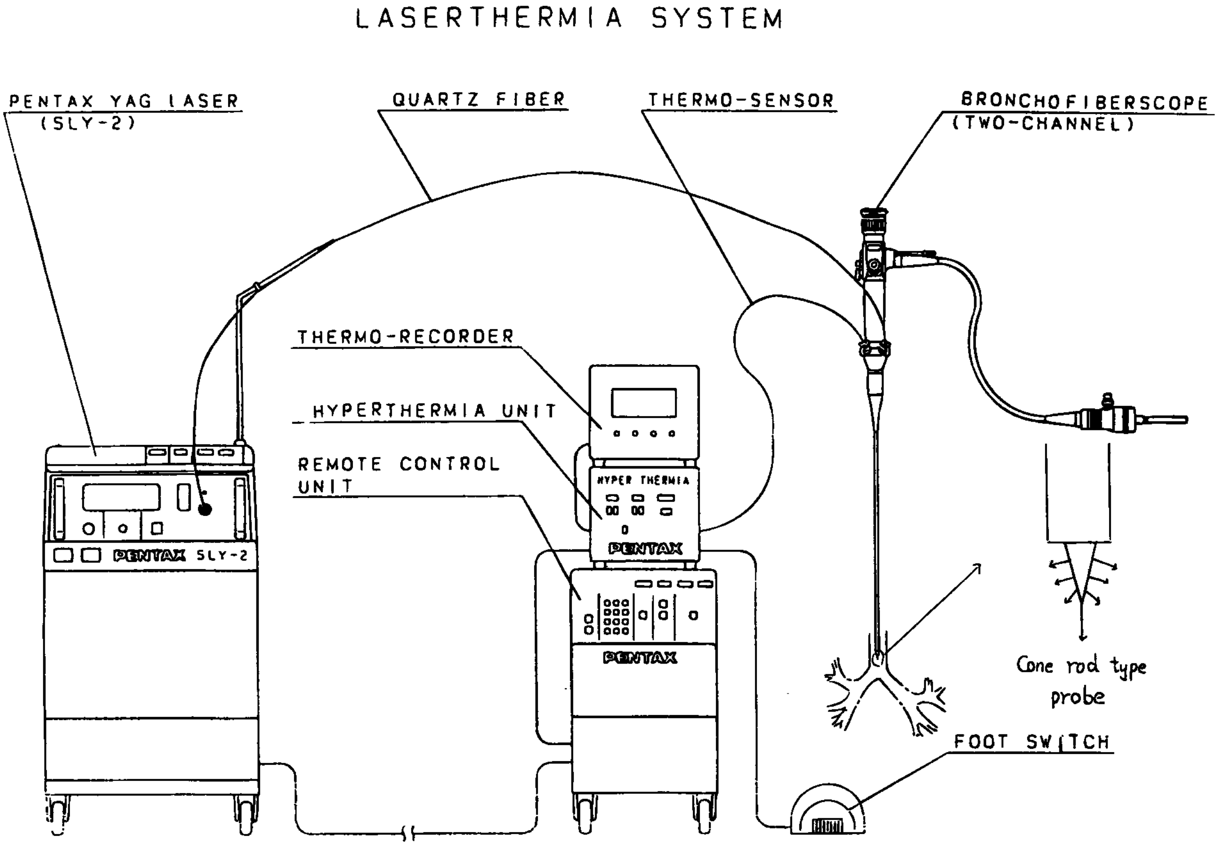

**Figure 1** Schematic Diagram of Laserthermia System

The results were categorized according to the therapeutic response after laser hyperthermia, subsequent behavior of the tumor, long-term results, and the current status of each patient recorded as of June 1992.

### Complete Response

Table 2 presents information on 9 patients who had a complete response after laser hyperthermia. Eight patients had squamous cell carcinoma, and the remaining patient (case 2) had an adenocarcinoma. In two patients (cases 1 and 6), the response was complete after a single course of treatment, whereas in three patients (cases 3, 4, and 5), a second laser hyperthermia session was required to achieve a complete response. For the remaining four patients (cases 2, 7, 8, and 9), a third laser hyperthermia session was necessary for a complete response. In these nine patients, there was no evidence of local recurrence, with a follow-up period ranging from 4 to 29 months (mean, 19.2 months). Three patients (cases 1, 2, and 6) who had a complete response subsequently died. One patient (case 6) died of a second lung cancer (small cell carcinoma). At postmortem examination, no evidence of residual cancer was found at the site of the first cancer. Two patients (cases 1 and 2) were found at autopsy to have evidence of widespread metastatic cancer, without evidence of recurrence at the treated site. The remaining six patients (cases 3, 4, 5, 7, 8, and 9) are still alive and disease-free with a follow-up ranging from 23 to 29 months (mean, 25.8 months).

Figure 2 shows the bronchoscopic findings of case 3 before and after laser hyperthermia. This case was a flat superficial tumor. In the first treatment, the probe was inserted at point (A) and the thermister was placed at point (B), and hyperthermia was performed for 30 minutes, whereas in the second 30-minute procedure, the probe was inserted in (B) and thermister was put at (A).

### Partial Response

Table 3 presents information on four patients with squamous cell carcinoma (cases 10, 11, 12, and 13) who had only a partial response to laser hyperthermia. They were given external beam radiation after laser hyperthermia failed to eradicate the cancer. As shown in Table 3, these four patients showed no local recurrence after a follow-up period ranging from 6 to 24 months (mean, 11.8 months). Two patients (cases 12 and 13) died of myocardial infarction, 7 and 10 months after laser hyperthermia, respectively. One patient (case 10) was found at autopsy to have evidence of widespread metastatic disease. However, there was no evidence of recurrence at the treated site in the right main bronchus. At the last follow-up, the remaining patient (case 11) was alive with no local recurrence, and the most recent bronchoscopic examination in this case showed no evidence of residual cancer.

### Roentgenographic Appearance and Tumor Size

Of the 13 cancers, 7 that were not visible on the chest roentgenogram showed a complete response to laser hyperthermia. The remaining 6 cancers were large enough to be detected on the chest roentgenogram. None of these lesions were associated with a complete response.

The overall surface area was estimated by the bronchoscopist at the time of the initial laser hyperthermia. However, the overall surface area of tumor occluding the bronchus could not be estimated correctly. There were two cases (6 and 8) with polypoid tumor in segmental bronchi that could be detected as secondary changes radiologically. Of the 9 cancers that were 3 $cm^2$ or smaller in surface area, a complete response was achieved in all. Of the 4 lesions that exceeded 4 $cm^2$ in surface area, none demonstrated a complete response.

### Complications

During the initial phase of the study, all patients reported the development of cough productive of blood-tinged sputum and gray necrotic material. This complication persisted for up to 2 weeks after treatment. Five patients had fever, pneumonia, excessive bronchial secretions, and mucosal plugging. Three patients experienced temporary airway obstruction that was caused by the necrotic debris. The necrotic debris was removed by flexible fiberoptic bronchoscopy. One patient (case 2) developed severe candidiasis at the treated situ on the tracheal wall. This patient died after an episode of hemoptysis caused by internal perforation. The postmortem examination showed residual necrotic debris and tracheal edema. No viable cancer cells were identified in the treated site in the tracheal wall. This patient had had previous cancer of a different histologic type and was found to have evidence of widespread metastasis of the first cancer.

### DISCUSSION

Laser hyperthermia is a new addition to standard treatment modalities such as surgical resection, radiation therapy, and chemotherapy for patients with bronchogenic carcinoma. The role of laser hyperthermia is limited to tumors within the bronchial wall because the depth of penetration of the activating light is no more than 2 cm and

**Table 2** Profile of patients who had a complete response after laser hyperthermia

| | | *Tumor* | | | *Power* | |
|---|---|---|---|---|---|---|
| *Case* | *Age, Sex* | *Cell type; Site* | *Surface area ($cm^2$)* | *No. of treatments* | *Density $W/cm^2$* | *Light Dose $J/cm^2$* |
| 1 | 60,m | Squamous; trachea | 2 | 1 | 2 | 2278 |
| 2 | 68,m | Adeno-carcinoma; | | 1 | 2 | 2223 |
| | | | 3 | 2 | 3 | 1790 |
| 3 | 70,m | trachea | | 3 | 3 | 2973 |
| | | squamous; | < 1 | 1 | 3 | 1497 |
| | | RB2 | | 2 | 2 | 1180 |
| 4 | 36,e | squamous; | 1–2 | 1 | 2 | 2441 |
| | | trachea | | 2 | 3 | 2223 |
| 5 | 69,m | Squamous; | 1–2 | 1 | 3 | 1648 |
| | | trachea | | 2 | 3 | 2535 |
| 6 | 66,m | Squamous; | < 1 | 1 | 3 | 1043 |
| | | LB10 | | | | |
| 7 | 68,m | Squamous; | | 1 | 2 | 3832 |
| | | trachea | 3 | 2 | 3 | 3181 |
| | | | | 3 | 3 | 2622 |
| 8 | 67,m | Squamous; | 2–3 | 1 | 3 | 3123 |
| | | LB8 | | 2 | 3 | 2932 |
| | | | | 1 | 3 | 1003 |
| 9 | 68,m | Squamous; | 3 | 2 | 3 | 1666 |
| | | RML | | 3 | 3 | 1324 |

Note. L.T. indicates laser hyperthermia; RB2, posterior segment of right upper lobe; LB10, posterior basilar segment of left lower lobe; LB8, anterior basilar segment of left lower lobe; and RML, right middle lobe.

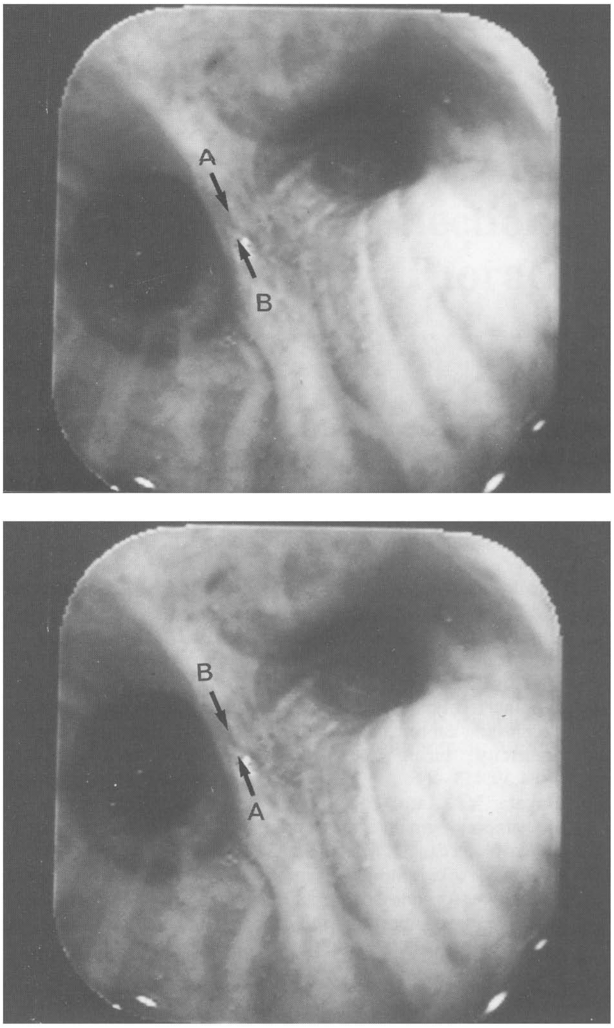

**Figure 2** Squamous cell carcinoma in the spur region of the right upper lobe bronchus before and after laser hyperthermia is probably much less than 1 cm. Therefore, the main application is in the treatment of carcinoma in situ or intramural invasive tumor. The likelihood of lymph node metastasis from these types of tumors is low (7, 8).

In our study, seven out of nine cancers with a complete response after laser hyperthermia were roentgenographically occult tumors less than 3 $cm^2$ in surface area and within the reach of the flexible fiberoptic bronchoscope. One patient (case 2) died of hemoptysis after treatment. This patient had a squamous cell carcinoma in the right lung that was resected, but afterward an adenocarcinoma was detected in the lower part of trachea. Laser hyperthermia was performed three times, obtaining complete response; however, the patient died of hemoptysis caused by perforation after 4 months. There was no evidence of recurrence at autopsy, but the death was considered to have caused by this treatment.

No other serious complications were experienced, apart from three cases of transient airway obstruction caused by necrotic debris. We recommend that a clean-up bronchoscopy should be performed to remove the secretions within 1 or 2 days after treatment, whether or not symptoms occur. Hayata and Kato et al (9) also routinely perform a clean-up bronchoscopy 1 or 2 days after HpD photodynamic therapy. They have reported excellent results in patients with early lung cancer. There have been few reports on laser hyperthermia for lung cancer (10).

We began to investigate local endoscopic thermal treatment for the application of contact Nd:YAG laser core rod type probe using low-grade capacity. Experiments on the normal dog bronchus using a local thermal beam showed extensive histologic changes in the submucosal layer. The thickness was parallel to the capacity, but there was little difference in submucosal change. We felt that it was proper for local thermal treatment to use low energy under 5 watts to have a good effect. Because upgrade of local tissue, thermal energy was counted on the point of 50°C in the center area under temperature control at 42.0°C ± 1.0°C at the point of 5.0-mm distance from the inserted probe, the thermister was placed 5.0 mm from the probe to confirm the temperature in operation. It was, necessary, therefore, to use a double-channel bronchoscope to insert the probe and thermister separately on the treatment site.

This was kept on up to 40°C after stopping the beam, but this required that a broader area be maintained or supported for a longer period under the temperature of 42.5°C for thermal treatment.

To date, nothing has been developed for local thermal restraint. This report is the first description of computer controlled local Nd:YAG laser hyperthermia. As a result of this investigation, a core rod-type probe power density is considered to be appropriate for local thermal treatment. Considering the thermal change by the cooling effect in living tissue organ, examination of beam's condition against the tumor is more effective in exploiting high-grade spreading of the contact terminal. Finally the development of an apparatus capable of easy clinical application and of providing an effective laser beam with a tissue thermometer are required.

### REFERENCES

1. Coosset JM, Dutreix J, Dufour J, et al. Combined interstitial hyperthermia and brachytherapy: Institute Gustave Roussy Technique and preliminary results. Int J Radiat Oncol Biol Phys 1984;10:307–312.
2. Emami B, Marks JE, Perez CA, Nusbaum GH, Leybovich, von Gerichten D. Interstitial thermoradiotherapy in the treatment of recurrent residual malignant tumors. Am J Clin Oncol 1984;6:699–704.

**Table 3** Profile of patients with a partial response after laser hyperthermia

| Case | Age, sex | *Tumor* Cell type: site | Surface area ($cm^2$) | No. of treatments | *Power* Density $W/cm^2$ | Light Dose $J/cm^2$ | Other therapy |
|---|---|---|---|---|---|---|---|
| 10 | 72,m | Squamous; | | 1 | 3 | 1854 | |
| | | R. main | 4 | 2 | 3 | 1534 | Radiation |
| | | | | 3 | 2 | 3177 | |
| 11 | 56,m | Squamous; | | 1 | 2 | 2353 | |
| | | R.U.L. | 5 | 2 | 3 | 3579 | Radiation |
| | | | | 3 | 3 | 3171 | |
| 12 | 62,m | Squamous; | | 1 | 3 | 1435 | |
| | | R.L.L. | 5 | 2 | 3 | 1794 | Radiation |
| | | | | 3 | 3 | 2034 | |
| 13 | 79,m | Squamous; | | 1 | 3 | 2301 | |
| | | R.U.L. | 5 | 2 | 3 | 2003 | Radiation |
| | | | | 3 | 3 | 2580 | |

Note.—R. main indicates Right main stem bronchus; R.U.L., Right upper lobe; R.L.L., Right lower lobe.

3. Aristizabal SA, Oleson JR. Combined interstitial irradiation and localized current field hyperthermia: results and conclusions from clinical studies. Cancer Res 1984;44:4757–4760.
4. Daikuzono N, Joffe SN. Artificial sapphire probe for contact photocoagulation and tissue vaporization with the Nd:YAG laser. Medical Instrumentation 1985;19:173–178.
5. Tajiri H, Oguro Y, Daikuzono N, Joffe SN. Experimental therapies of human pancreatic carcinoma transplanted to nude mice: a study on photodynamic and local interstitial hyperthermia using low power Nd:YAG laser. Proceedings of SPIE; The International Society for Optical Engineering 712: Lasers in Medicine 1986;22–26.
6. Suzuki S, Narumi H, Aoki J, Miwa T, Daikuzono N, Joffe SN. Experimental studies of endoscopic local hyperthermia with Nd:YAG laser (Laserthermia) in Laser Surgery: advanced Characterization, Therapeutic, and System Proceedings of SPIE; The International Society for Optical Engineering 1989;1066:225–230.
7. Cortese DA, Pairolero PC, Bergstralh EJ, et al. Roentgenographically occult lung cancer; a ten-year experience. J. Thorac Cardiovasc Surg. 1983;86:373–380.
8. Woolner LB, Fontana RS, Cortese DA, et al. Roentgenographically occult lung cancer: pathologic findings and frequency of multicentricity during a 10-year period. Mayo Clin Proc 1984;59:453–466.
9. Hayata Y, Kato H, Konaka C, Ono J, Takizawa N. Hematoporphyrin derivative and laser photoradiation in the treatment of lung cancer. Chest 1982;81:269–77.
10. Suzuki S, Aoki J, Shiina Y, Nomiyama T, Miwa T. New ceramic endoprobes for endoscopic contact irradiation with Nd:YAG laser: experimental studies and clinical applications. Gastrointestinal Endoscopy 1986;32:282–286.

